# Guidance for a clinical and forensic diagnosis of pediatric abusive head trauma: a narrative review

**DOI:** 10.3389/fped.2026.1706089

**Published:** 2026-02-13

**Authors:** Monica Concato, Anita Galic Mihic, Tijana Petrovic, Djordje M. Alempijevic, Davide Radaelli, Stefano D'Errico

**Affiliations:** 1 Department of Medical Surgical and Health Sciences, University of Trieste, Trieste, Italy; 2 Institute of Forensic Medicine and Criminalistics, School of Medicine, University of Zagreb, Zagreb, Croatia; 3 Faculty of Medicine, Institute of Forensic Medicine, University of Belgrade, Belgrade, Serbia

**Keywords:** abusive head trauma (AHT), clinical diagnosis, forensic diagnosis, non-accidental head injuries (NAHI), Shaken Baby Syndrome

## Abstract

The term Shaken Baby Syndrome, now largely replaced by the more general term abusive head trauma (AHT), poses significant challenges for forensic assessment and investigation. The clinical assessment of these cases remains a complex process that necessarily involves collecting a complete medical history, taking into account the caregivers and the child's socio-economic and family context, a thorough physical examination, and additional diagnostic investigations. In cases where the child has died, the autopsy—whether performed for investigative or purely diagnostic purposes—remains an essential step requiring a rigorous methodological approach, technical expertise, and in-depth knowledge of the subject. Guided by previously conducted instrumental examinations, the autopsy includes a fundamental macroscopic evaluation followed by the essential histological assessment of the injuries. Given the importance of the topic, and the implications of an erroneous conclusion for both the young patient and their family, this work aims to compile methodological updates from the last ten years, in order to promote greater uniformity in the medico-legal practice.

## Introduction

Research on a topic as complex as abuse of children immediately faces multiple methodological and historical challenges, arising not only from differences in diagnostic approaches but also from variations in definitions and terminology. The medicolegal hypothesis of Shaken Baby Syndrome (SBS), in use since the early 1970s (1), stated that violent shaking of an infant could cause a triad of pathological findings—subdural hemorrhage, retinal hemorrhages, and brain damage—that, in the absence of plausible explanations, could reliably be attributed to non-accidental trauma. Initially accepted as an automatic diagnostic interpretation, this hypothesis became the subject of intense scientific debate, as the term “*shaking*” alone failed to encompass the full range of injury mechanisms (1–6).

Additional terminology was subsequently introduced, including non-accidental head injuries (NAHI) - in the early 2000s following recommendations by the American Academy of Pediatrics and the UK Crown Prosecution Service (1) - and abusive head trauma (AHT) as later proposed by the Centers for Disease Control and Prevention (7). The development of terminology arises from the fact that the findings of the triad can indeed be caused by shaking, but they may also be completely independent of it (8), and from the additional observation that, although shaking alone has the potential to cause neurological damage, blunt impact or a combination of shaking and blunt impact may result in significant neurological injuries also (7).

Even from an initial preliminary search, it is evident that authors in the literature often use the terms interchangeably, essentially referring to the same phenomenon. This, for instance, complicates the conduct of robust epidemiological research capable of providing clear evidence of the problem.

Several studies refer to an incidence ranging from 14 to 40 cases per 100,000 children in industrialized countries (9), while others report 29.7/100,000 person-years in children less than one year (7). AHT represents the leading cause of death among children under the age of two (7) and the most common cause of disability in children who are victims of physical abuse (1). Approximately 1 in 4 children affected dies, and more than half suffer severe neurological injuries and/or permanent visual impairments (1). However, due to a lack of updated statistical data (9) and the considerable challenges associated with diagnosis (7), it is suspected that the phenomenon is significantly underreported.

Excessive or inconsolable crying, observed in about 20% of infants, especially between the second week and the sixth month of life, appears to be a significant trigger in the abuse of newborns and infants, as it can have a major impact on caregivers (7, 9, 10). During the anamnesis, potential individual risk factors related to the infant must be investigated, such as male infants, prematurity, and multiple births (1, 11). Additionally, risk factors related to the perpetrator are considered, including the family's socioeconomic context (1), social isolation, lack of adequate support structures, and poverty (7). Among all these, the most significant biological risk factor lies, for some authors, in the considerable disproportion between the size and weight of the infant's head compared to the rest of the body. This, combined with poor head and trunk control, the fragility and immaturity of the brain tissue, and greater vulnerability of neonatal/infant bridging veins, makes the cerebral parenchyma more susceptible to damage due to incomplete axonal myelination (9, 12, 13).

The spectrum of head injuries grouped under the term AHI includes impact with a stationary object, direct blows to the skull, and acceleration-deceleration forces (2, 14), often resulting from shaking the child, typically while holding the chest or limbs. Forceful and repeated shaking is the primary mechanism reported by perpetrators during confessions, while an actual impact is reported in only 24% of cases (15). The energy generated by shaking results in sudden acceleration and deceleration of the brain around the cervical neuroaxis, causing brain movement, rotation, and bouncing against the hard structure of the skull base (1).

Given the complexity of the subject, which leads to a challenging diagnostic process, this review aims to provide a comprehensive overview of current knowledge, highlighting gaps and supporting the scientific community in improving diagnostic accuracy.

## Methods

To promote uniformity in the clinical and medico-legal diagnostic practice of AHT, this review explores the theoretical and practical advances proposed in the literature over the past 10 years. Building on this foundation, it discusses the current needs of professionals who must personally face the challenges associated with a complex diagnostic process. The search, conducted in PubMed in December 2025 using the terms “Non-Accidental Head Injury”, “Shaken Baby Syndrome”, “clinical diagnosis”, “medico-legal diagnosis” and “forensic diagnosis”, yielded a total of 149 results. Two authors independently screened and discussed the results. A first selection was made using only titles and abstracts, excluding all works that were irrelevant to the study's objective, while in a later phase the focus was placed on concrete new contributions to the diagnostic process in both clinical and forensic practice, with emphasis on the last 10 years. Therefore, 44 studies were finally selected and discussed below, as shown in Table 1 (3–8, 11, 15–50).

**Table 1:** Articles included in the review.

N°	Article	Contribution
1	(16)	Clinical follow-up
2	(17)	Clinical follow-up
3	(18)	Hemosiderin deposits in the eye in the evaluation of repeated trauma
4	(19)	Nomenclature changes
5	(20)	Examination of the dura mater at the autopsy table
6	(3)	Inclusion of spinal injury assessment both in MRI and in pathology
7	(21)	Italian recommendations for clinical diagnosis
8	(22)	Includes spinal injuries as an integral component of the characterization of SBS-related injuries
9	(23)	Histology of ocular lesions
10	(67)	Retinal hemorrhages dating
11	(25)	The superior spatial resolution on SWI sequences better identifies microhemorrhages compared with T2* GRE
12	(4)	Ret Cam in retinal hemorrhages, differential diagnosis
13	(26)	Diagnostic uncertainty
14	(27)	Dating brain lesions at MRI
15	(28)	Subdural fluid collections as key neuroimaging findings, dd acute and chronic trauma
16	(29)	Retinal hemorrhages diagnosis
17	(30)	Clinical prediction rules
18	(31)	Includes spinal injuries as an integral component of the characterization of SBS-related injuries
19	(7)	Susceptibility weighted imaging (SWI) in the detection of cerebral microhemorrhage at the early stage as prognosis predictor
20	(5)	Clinical prediction rules as support in diagnosis
21	(32)	OCT
22	(33)	OCT
23	(34)	Use of MRI in the diagnosis
24	(35)	Retinal hemorrhages diagnosis and follow-up
25	(36)	High-speed video imaging could as a valuable tool for evaluating accidental fall
26	(37)	Update in guidelines
27	(11)	Considering risk factors in the anamnestic phase
28	(38)	Against uncritical application of diagnostic tests or of the triad
29	(6)	Critical al circular reasoning
30	(39)	Differential diagnosis
31	(40)	New technologies as tools for event identification
32	(41)	The role of hematomas as a sentinel sign
33	(42)	Differential diagnosis in bone fractures
34	(43)	Clinical prediction tool to calculate the probability of AHT
35	(44)	Differential diagnosis for RH
36	(45)	Differential diagnosis
37	(46)	Differential diagnosis for RH
38	(47)	Differential diagnosis
39	(53)	The role of the triad
40	(48)	Differential diagnosis
41	(49)	Differential diagnosis
42	(56)	Artificial Intelligence
43	(8)	Differential diagnosis
44	(50)	Isodense and hypodense subdural hematomas in repeated trauma

MRI, magnetic resonance imaging; SBS, shaken baby syndrome; SWI, susceptibility weighted imaging; GRE, gradient echo; OCT, optical coherence tomography; AHT, abusive head trauma; RH, retinal haemorrhage.

## Results and discussion

Prior to the discussion, it is necessary to clarify the terminology used in the literature search. Although the most authoritative societies have long recommended abandoning the term SBS, it was considered appropriate to include it in the initial phase in order to avoid the potential loss of relevant content. In the present manuscript, the term abusive head trauma has been preferentially used throughout the text, in accordance with the most recent and widely accepted terminology (19), in order to ensure terminological consistency and uniformity.

A misdiagnosis in cases with suspected abusive head trauma can have significant consequences both for the lives of the children and for those of their parents. An accurate evaluation of the injury pattern is critical in suspected cases of abuse, bearing in mind that the typical intracranial triad—subdural hematoma, retinal hemorrhages, and encephalopathy-—may have alternative causes, including accidental trauma and certain medical conditions (22). This underscores the need to develop, based on the knowledge currently available to the clinical and forensic community, international guidelines to guide diagnosis.

### Recommendations for clinical diagnosis

From a clinical perspective, the initial signs and symptoms that may raise diagnostic suspicion of non-accidental head injuries are heterogeneous. In more severe cases, if death has not occurred, the infant may present clinical signs of serious neurological damage that require immediate treatment (seizures, severe alterations of consciousness, respiratory pauses, bradycardia, decreased alertness, severe apnea, upward eye deviation, signs of increased intracranial pressure or herniation), either described by the parents or observed by healthcare providers. Conversely, in less severe cases, the child may present with nonspecific signs (crying, irritability, changes in sleep or feeding habits, fewer smiles, vomiting, respiratory pauses, pallor, suspected pain) that can lead to misdiagnosis (1). Narang et al. (41), however, emphasizes the importance of not underestimating certain sentinel injuries such as bruises—lesions that are often missed by examiners in cases of abuse (41). Nevertheless, it is important that even when evaluating simple symptoms, within the context of the full clinical picture, they can at least raise a suspicion of AHT, prompting the need for further investigations (26).

Prior to beginning clinical investigation, medical and non-medical history is of critical importance. History provided by the family or the caregivers, a delay in seeking medical care, the absence, implausible or inconsistent explanations for the observed clinical signs, references to minor head trauma or any history of trauma, and mention of episodes of inconsolable crying may in fact guide the diagnosis (1). Patient- and environment-related risk factors must be considered, while recognizing that a risk factor does not imply causation, as Laurent-Vannier et al. (11) emphasizes (11).

AHT cases are often characterized by extra-axial intracranial hemorrhages—subdural hemorrhage in approximately 72%–93% of cases, sometimes combined with subarachnoid hemorrhage. They are generally multifocal, bilateral, and do not produce a mass effect, but instead span the convexity of the cerebral hemispheres and accumulate in the longitudinal fissures and along the tentorial attachment. Particularly suggestive is the presence of hemorrhage along the falx cerebri and subdural collections in the posterior cranial fossa (1).

In addition to the injuries described above, the most recent literature also considers the assessment of cervical spinal damage (22, 31), which is detected on MRI in 15%–78% of cases (31), proposing its inclusion in the characterization of shaking-related injuries. In particular, it focuses on evaluating the presence of subdural and epidural hematomas at the cervico-medullary junction, as well as injuries to the nuchal, atlanto-occipital, and atlanto-axial ligaments — typical lesions caused by a flexion mechanism (22). Moreover, when abuse is suspected, evaluation of the entire length of the spinal cord should be undertaken. Spinal hemorrhagic collections, caused by injury to the radicular veins, though still poorly studied, seem highly suggestive of abuse (3).

Retinal hemorrhages are not always present in AHT cases. Their interpretation varies according to their location within the eye, laterality, appearance, size, and shape. Based on their number and extent, they are classified into three types, as shown in Table 2 (1).

**Table 2:** Classification of retinal hemorrhage.

Type	Hemorrhage shape	Location	Other characteristics
Type 1	Flame-shaped, blot-like, or dot	Intraretinal hemorrhages in the posterior pole	
Type 2	Small, dome-shaped	At the posterior pole, around the optic disc or periphery	Alone or in combination with Type 1
Type 3	Profuse, multiple	Intra-, pre-, or subretinal, covering the entire retina or spreading to the periphery	Often combined with unilateral or bilateral macular hemorrhagic plaques;

While it was common practice to consider type 3 retinal hemorrhages pathognomonic of AHT especially when seen in combination with subdural hemorrhage, massive cerebral edema, or skeletal injuries that strongly indicate abuse (1), the most recent literature reports that no single pattern or distribution of retinal hemorrhages is entirely specific for the diagnosis of cases of abusive head trauma (29, 51). However, it is emphasized that in the presence of intraocular findings such as retinal hemorrhages — particularly if preretinal and subretinal, peripheral and/or intrascleral — and in the absence of alternative diagnoses, the diagnosis of AHT may be established with a high degree of confidence (29). Other ocular findings in children with suspected abusive head trauma may include vitreous and choroidal hemorrhages, papilledema secondary to increased intracranial pressure and infraorbital hemorrhages (involving the sclera, optic nerve sheath, extraocular muscles, or orbital fat).

The expertise of an ophthalmologist is always required for the evaluation of the intraocular findings (32) as the degree of intracranial trauma and the severity of the neurological injury correlate with the extent of the intraocular hemorrhages. High-resolution imaging by optical coherence tomography (OCT) has been explored in the evaluation of retinal injuries (33) even if data remain limited due the lack of availability of the technology (32). Among the new diagnostic methodologies, as an alternative to the common fundus examination, Elinder er al (4). proposes the RetCam, which also allows subsequent observers, not familiar with the specific details of the case, to evaluate the findings (4). In their report, Ksiaa et al. (35) describe for the first time the use of swept-source optical coherence tomography (SSOCT) for the diagnosis and monitoring of retinal pathology in children affected by abusive head trauma. OCT reveals vitreous and retinal alterations that are not detectable on clinical examination and have never been previously reported. Moreover, the examination proves useful in follow-up, as retinal atrophic changes may become apparent once retinal hemorrhages have resolved (35).

Lastly, reference should be made to fractures as part of the other lesions. Limb, rib and skull fractures are the most characteristic findings. Rib fractures are typically multiple, contiguous and symmetrical and are more commonly found at the posterior arches and costovertebral junction. Multiple or bilateral skull fractures, primarily located in the occipital area, as well as fractured crossing suture lines, are frequently associated with non-accidental head trauma (1).

### Imaging

CT scan—for bone and soft tissue windows, and MRI of the brain and cranial region are essential in the diagnosis of AHT (1), with MRI considered the superior technique in assessing brain lesions (34). Alongside the recommended techniques—spin echo sequencing, diffusion-weighted imaging, and fluid-attenuated inversion recovery sequencing-, susceptibility-weighted MR imaging (SWI) has recently been used as a prognosis predictor through the detection of cerebral micro-hemorrhage at the early stage (7) thanks to its higher spatial resolution (25). It is important to note, however, that the most recent literature also includes the study of the spinal cord through magnetic resonance imaging, as these lesions play a supportive role in the diagnostic process (3).

Subdural fluid collections are key neuroimaging findings in AHT (28). Hahnemann et al. (28) defines the so-called Mixed Appearance Pattern of these rarely unifocal lesions, as they may present as subdural hematomas, subdural hygromas, subdural hematohygromas—most frequently –, and chronic subdural hematomas (28, 50). More recent studies also focused on the imaging of extensive brain lesions regarding the parenchyma of the brain caused by shaking-related mechanisms (52).

Radiological investigations represent an important tool not only in the identification of the lesions but also in their dating. Although reliable reference studies in this field are complex—due to the need for confirmation of suspected abusive head trauma and the timing of injury—and further research is required, some authors highlight the importance of the information provided by imaging (27). In their study, hyperdensity on CT scans was observed up to 9 days post-injury, which is partially in agreement with previous studies. Membranes were detected as late as 283 days on CT scans, while MRI findings appeared as early as 2–4 weeks (27). In addition to the assessment of known subdural membranes, the tadpole sign,—a round- to oval-shaped blood clot within or adjacent to an expanded bridging vein -, has recently been introduced as an additional MRI finding, appearing between day 0 and day 11 and day 0 to day 46 depending on the sequences used, and considered an early phenomenon in AHT (27).

Although neuroimaging alone cannot precisely date lesions, or prove repeated trauma, it offers a time interval that must be integrated with the anamnesis and the clinical findings (25, 28, 50). While further studies would be necessary, hemosiderin deposition on membranes could contribute to estimating the age of the injury when observed on MRI (25). Lastly, with regard to fractures and their dating, a follow-up radiograph should be performed approximately two weeks after the initial evaluation—even with preliminary negative results—since recent fractures may not become apparent until 7–10 days post-trauma (1).

### Differential diagnosis

An assessment that cannot be overlooked should concern the differential diagnosis of AHT. Without it, no case evaluation should be considered complete, and it represents one of the greatest challenges for professionals, who often find themselves working in contexts where certain information is lacking or cannot be obtained. Before moving on to further considerations, it is necessary to underline the well-known debate on the validity of the triad and the fact that diagnosis cannot be based solely on the presence of subdural and retinal hemorrhage and encephalopathy, nor can it disregard a thorough investigative protocol (8, 53, 54).

Unlike earlier studies, the most recent literature gives considerable attention to alternative diagnoses (48), including numerous genetic conditions that can cause bleeding in the encephalic region and therefore be mistaken for AHT (49). It is fundamental to consider all hypotheses, not overlooking either the existence of benign conditions such as benign external hydrocephalus (45), or congenital or predisposing conditions (49) such as, for example, vascular malformation (47), nor the possibility of non-accidental injury (4, 47, 49). Literature identifies several conditions which can cause both RH and SDH (4) outlining a considerable complexity of the scenario.

Highlighting certain aspects, from a clinical point of view, macrocephaly or an increase in cranial circumference must be taken into consideration by obtaining all available measurements starting from birth, as well as those of the parents (39). Indeed, they represent an alternative diagnosis in the presence of the triad, raising the possibility of a medical explanation (39). Also with regard to retinal haemorrhage, the literature has questioned the unequivocal correlation between the pathognomonic nature of retinal lesions and retinoschisis in AHT (46, 55). In fact, such lesions may be observed in a wide range of conditions beyond AHT, including infections and sepsis, increased intrathoracic pressure caused by choking or coughing, haematological diseases such as leukaemia (44), or accidental injuries (46). Even in fracture-related injuries, alternative causes are investigated, one example being a predisposition to increased bone fragility, which is typical, for instance, of individuals with Alagille syndrome (42).

### New frontiers

The most recent sources describe the use of screening tools for clinical prediction including Pediatric Emergency Applied Care Research Network (PECARN), Canadian Assessment of Tomography for Childhood Head Injury (CATCH), and Children's Head Injury Algorithm for the Prediction of Important Clinical Events (CHALICE), as valuable tools in the management of pediatric head injuries, although they were not specifically developed for abused patients (30). Alongside this, artificial intelligence has shown promising results in distinguishing between accidental and non-accidental trauma (56).

To standardize the workflow and improve diagnostic accuracy—based on the premise that intuition, clinical experience, and pathophysiological knowledge alone are not sufficient to make clinical decisions—there is a growing shift toward the use of clinical prediction rules, such as PredAHT (5, 43) or the PediBIRN-7, valuable support tools for strengthening clinician confidence in their impressions and for confirming or excluding pending decisions (5), especially in combination with a comprehensive multidisciplinary assessment (43).

Moreover, focusing on the most recent technologies, inertial measurement unit (IMU)-based detection systems enhanced with machine learning have been proposed as an aid in detecting and classifying harmful shaking events. More broadly, the combination of sensor-based systems with artificial intelligence holds potential both as an assessment tool and as a predictive instrument for early identification of risk (40).

A critical and cautious approach in suspected AHT cases is essential, following the reasoning of Wester (57) and considering the methodological critique by Lynøe and Eriksson (38). The former (57) concerns the consensus statement published in 2018 by the working group of Choudhary et al. (2) and its subsequent adoption within the Norwegian medical community (58). Lynøe and Eriksson (38) emphasize that no diagnostic test alone can definitively “prove” abuse, and that assumptions combined with diagnostic criteria may introduce bias (6). The blind application of a concept, in fact, may limit the consideration of alternative differential diagnoses (38).

Given the severe outcomes, which translate into very high mortality and significant disability among survivors of AHT (16, 17), even at a very young age and within a short interval from the suspected abusive event (17), the most recent literature highlights the importance of follow-up for survivors of AHT aimed at developmental assessment and the early coordination of rehabilitative services whenever possible (16, 17).

### Recommendations for forensic diagnosis

Especially nowadays, when newborns undergo thorough screening tests, a negative result at birth generally implies the exclusion of perinatal lesions, whereas unexplained findings, on the contrary, direct the investigation towards possible suspected abusive practices (34). It is precisely in this context that, prior to conducting additional examinations, it becomes crucial to rely on a comprehensive review of all available medical documentation, which must necessarily be examined alongside the testimonies provided by parents or caregivers.

In fatal cases of non-accidental head injuries, whether investigative or purely diagnostic, the autopsy represents an indispensable moment of study that requires a rigorous methodology, the application of technical skills at the autopsy table, and expertise in interpreting macroscopic and microscopic findings (1). Performing both a total body radiographic exam and a CT scan before the autopsy is considered essential to verify congenital and/or potentially traumatic pathological findings. These may direct or modify the forensic pathologist's strategy at the autopsy table (1).

In cases of suspected or presumed abusive head trauma, examination of the brain is fundamental. Fixation of the whole organ in formalin allows for the choice of different cutting techniques enabling better observation of the pathological findings, which must be described in terms of location, size, colour characteristics (1). A valid aid in the evaluation of subdural pathology while performing the autopsy, could be the use of the optical transparency of the dura mater with glycerol as proposed by Cheshire et al. (20). It allows for better visualization and assessment of even minimal haemorrhagic spread, with the advantage of obtaining a clearer and more detailed photographic description and documentation of subdural hematomas, free from any artefacts caused by procedures performed on the autopsy table (20). As in the living patient, Colombari et al. (3) suggest including spinal cord evaluation at the autopsy table in the challenging diagnosis of AHT. The presence of spinal injuries identified by the pathologist would, in fact, support the diagnosis of suspected non-accidental injury with a higher level of certainty (3).

Histological examination is a crucial further step and must evidently be part of the pathologist's knowledge and skills, no longer delegable to other professionals. The microscopic examination of the brain, both through standard or special stains and immunohistochemical techniques, provides evidence of early brain damage as well as a reasonably accurate dating of the haemorrhagic findings typical of AHT or brain-cell injury (1). Not only important for assessing brain injury, histology also proves valuable in determining the timing of retinal haemorrhages and retro-ocular tissues, assuming a role of fundamental importance in forensic medical practice (23). As underlined by multiple authors (59–61), immunohistochemistry is of particular importance also in the study of retinal haemorrhages, especially in cases where there is poor erythrocyte representation or lysis of erythrocytes in the absence of early hemosiderin deposits detectable by Perls’ histochemical staining (59). As highlighted by Plowey et al. (61), it not only provides new diagnostic insights but also helps prevent misinterpretation due to post-mortem artifacts, such as swelling of Müller cell foot processes, which can mimic retinal haemorrhage (61).

The dating of hemorrhagic lesions continues to be a focus of study and represents one of the more difficult aspects in medicolegal proceedings, and a valuable aid can be sought precisely in microscopic and immunohistochemical evaluation as underlined in Table 3 (1).

**Table 3:** Histological stains (H) and immunohistochemical markers (IHC) employed in evaluation of the injuries (1, 59–61).

Tissue	Technique	Stain/markers	Findings	Notes
Skin	H	H&E	Hemorrhage	Aids in dating hemorrhagic lesions
Bones	H	H&E	Fractures	Aids in dating hemorrhagic lesions
Retina	H	H&E	Hemorrhage	Aids in dating hemorrhagic lesions
		Perls's	Hemorrhage	Aids in dating hemorrhagic lesions
	IHC	Glycophorin A and C	Intratissue hemorrhagic extravasation	Aids in dating hemorrhagic lesions
		ubiquitin	Markers of axonal injury affecting the retinal nerve fiber layer	Experimental use
		GFAP	Reactive gliosis	
		Beta-APP	Markers of axonal injury affecting the retinal nerve fiber layer	
		Nestin	Damage in retinal tissue	
		CD44	Damage in retinal tissue, High specificity for AHT cases.	
		Collagen type IX		
Spine	H	H&E	Spinal cord injuries, injuries of the atlanto-occipital or cervicodorsal junction	
Extra-axial space	H	H&E	Subdural and subarachnoid hemorrhage	Aids in dating hemorrhagic lesions
		Perls	Hemosiderin deposits	Aids in dating hemorrhagic lesions
	IHC	Glycophorin-A	Hemorrage	Aids in dating hemorrhagic lesions
Cerebral parenchyma	H	H&E	Neuronal degeneration, immediate nuclear shrinkage, axonal and nuclear swelling, apoptosis, blood vessels congestion and vessels proliferation	
	IHC	β-Amyloid Precursor Protein (β-APP)	Detects early axonal damage	Aids in dating hemorrhagic lesions
		GFAP (Glial Fibrillary Acidic Protein	Identification of reactive astrogliosis	
		CD3	Documents lymphocytes T infiltration	
		CD68	Documents macrophage infiltration	

In their retrospective study, Delteil et al. (24) addressed the dating of both subarachnoid hemorrhage and retinal hemorrhages by applying simple and reproducible criteria. In particular, the latter is considered to be of limited reliability for estimating the timing of injury (18, 24) highlighting the need for further research (18). Furthermore, the application of autopsy and histological findings not only enhances diagnostic accuracy and supports scientific conclusions for differentiating accidental from non-accidental causes of head trauma, but also supports medico-legal evaluation, including expert witness testimony and the credibility of evidence in legal proceedings. This highlights the relevance of these methodological and technical advances beyond the laboratory or autopsy room.

Ultimately, with regard to the search for alternative causes and the study of the dynamics of events, although forensic biomechanics cannot always be applied in crime scene investigation due to the countless unknown variables, an interesting insight is provided by Kunz et al. (36). In their study, they indicate that high-speed video imaging could serve as a valuable tool for evaluating falls. Applying this conclusion more broadly, one could consider, in the future, a practical use of this technology in general for head injuries (36).

### Guidelines

In clinical as well as in forensic practice, guidelines, best practices, and recommendations are essential foundations in daily professional activities. And it is precisely in this field that, especially today, it remains difficult to identify univocal references supported by global consensus, making an already complex diagnosis even more uncertain.

At the global level, various attempts have been made to establish a consensus within the scientific community, the most recent of which was published by Choudhary et al. (2) in May 2018. It was developed through the contribution of as many as 15 major national and international professional medical societies and organizations (2) and it can be accessed on the website of the National Center on Shaken Baby Syndrome (https://www.dontshake.org/learn-more). The same source also relies, among others, on the American Academy of Pediatrics (2020) (41), the Canadian Paediatric Society (62) and the French Society of Physical Medicine and Rehabilitation (11).

Dating back back to 2011 is the attempt of the French Society of Physical and Rehabilitation Medicine to issue clinical guidelines in order to define a useful and reliable diagnostic algorithm for suspected AHT cases (1, 63). Those recommendations for good care practices were later revised in 2017 (64), emphasizing the documented injuries and the clinical history provided by adults, rather than subjective elements (37). The latest update of the French guidelines, however, underlines the need to involve a multidisciplinary team and the importance of differential diagnoses (65). The level of diagnostic certainty is defined not based on risk factors, but on the explanations provided and the type of lesions objectively observed (51, 63, 66) as shown in Table 4.

**Table 4:** The French guidelines, in the 2011 edition and the 2017 update, should be noted, in particular for the change regarding retinal hemorrhages, which are no longer classified into different types.

Level of suspicion	Findings	2011 guidelines	2017 guidelines	Notes
Highly Likely	Extra-axial Hemorrhages	Multifocal (subdural, subarachnoid)	Multifocal with clots on the convexity (vertex), indicative of bridging vein rupture; or multifocal associated with retinal hemorrhages of any type; or unifocal with cervical and/or spinal cord injuries	Consistent with hypoxic brain injuries, cervical/spinal cord injuries, fractures, bruising, and reports of violent shaking by a witness
	Retinal hemorrhages	Type 3 RH	Any type	
	Clinical history	Absent, inconsistent, changing over time, or incompatible with injuries or child's age	Fluctuating or inconsistent clinical history with the observed lesions or the child's age, and after ruling out differential diagnoses	
	Additional findings	Hypoxic brain injury; cervical spinal injuries (e.g., cord hematoma, atlanto-occipital dislocation); eyewitness account of violent shaking	\	
Probable	Extra-axial hemorrhages	Multifocal SDH (±retinal hemorrhages of any type) OR unifocal SDH in the presence of Type 2 or 3 RH	Multifocal, even in the absence of other lesions; or unifocal with intraretinal RH limited to the posterior pole;	
	Retinal hemorrhages	Type 2 or 3 RH	or RH affecting the retinal periphery and/or multiple layers, either unilateral or bilateral.	
	Clinical history	Absent, inconsistent, changing over time, or incompatible with injuries or child's age	fluctuating or inconsistent clinical history with the observed lesions or the child's age, and after ruling out differential diagnoses	Consistent with accidental head trauma.
Possible	Extra-axial hemorrhages	Unifocal SDH	Not specified	
	Retinal hemorrhages	Not specified	Not specified	
	Clinical history	Absent, inconsistent, changing over time, or incompatible with injuries or child's age	Not specified	
	Additional Findings	In cases of unifocal SDH with RH limited to the posterior pole (type 1), there is no consensus on whether it should be considered probable or possible	\	
Excluded	Extra-axial hemorrhages	unifocal SDH with adjacent contusions and linear skull fracture	Unifocal SDH, with impact marks, unilateral or contralateral, compatible with the alleged mechanism: scalp contusion and possibly a linear fracture in the corresponding area	
	Retinal hemorrhages	None or consistent with trauma		
	Clinical history	Consistent over time, compatible with the injuries and child's age, and describes accidental or violent head trauma	Consistent clinical history	

RH, retinal haemorrhage; SDH, subdural haemorrhage.

Both the Italian Society of Neonatology (SIN) (https://blog.sin-neonatologia.it/wp-content/uploads/2020/06/SHAKEN-BABY-LA-SINDROME-DEL-BAMBINO-SCOSSO.pdf) and the Italian Society of Pediatric Emergency-Urgency Medicine (SIMEUP) (https://simeup.it/wp-content/uploads/2022/10/OIRM_201706_Protocollo-trauma-cranico-2017.pdf) (21) as well as the Italian Coordination of Services against Child Abuse and Neglect (https://cismai.it/sindrome-del-bambino-scosso/) or Bambino Gesù Pediatric Hospital (ospedalebambingesu.it), have published information and recommendations on Shaken Baby Syndrome. Da Dalt et al. in particular (21) explored in 2018 the management and disposition of children presenting to the emergency department with head trauma, within the first 24 h of their injury, although excluding the evaluation of spinal lesions (21). Therefore, even at the national level, although various organizations have contributed over time particularly to the clinical diagnosis of AHT, further efforts are needed in order to standardize both clinical and forensic practice.

## Conclusions

The diagnosis of AHT remains a complex task for professionals involved in suspected cases. Over the past decade, several advances have emerged, driven in part by technological progress and greater knowledge in radiological techniques. Current approaches increasingly move beyond reliance on the triad alone, proposing instead a multifactorial diagnostic model that requires interdisciplinary collaboration among specialists—including clinicians, radiologists, forensic physicians, and pathologists. Such cooperation is important both in routine practice and in the development of a shared, continuous education on the topic.

Clinical guidelines are often limited and, when available, are frequently fragmented across national contexts, further complicating the diagnostic process. References to best practices in forensic activities are even more limited and remain sparse, resulting in gaps in the practical management of these cases.

This lack of harmonization across national and international guidelines emphasizes the need for closer cooperation within the scientific community. It should encourage collaboration among professionals and scientific societies, ultimately leading to the development of internationally standardized diagnostic criteria that integrate clinical, radiological, and forensic evidence.
